# *Epilobium angustifolium* L. Essential Oil—Biological Activity and Enhancement of the Skin Penetration of Drugs—In Vitro Study

**DOI:** 10.3390/molecules26237188

**Published:** 2021-11-26

**Authors:** Anna Nowak, Wiktoria Duchnik, Edyta Makuch, Łukasz Kucharski, Paula Ossowicz-Rupniewska, Krystyna Cybulska, Tadeusz Sulikowski, Michał Moritz, Adam Klimowicz

**Affiliations:** 1 Department of Cosmetic and Pharmaceutical Chemistry, Pomeranian Medical University, PL-70111 Szczecin, Poland; lukasz.kucharski@pum.edu.pl (Ł.K.); adklim@pum.edu.pl (A.K.); 2 Department of Pharmaceutical Chemistry, Pomeranian Medical University, PL-70111 Szczecin, Poland; wiktoria.duchnik@pum.edu.pl (W.D.); michal.moritz@pum.edu.pl (M.M.); 3 Department of Chemical Organic Technology and Polymeric Materials, Faculty of Chemical Technology and Engineering, West Pomeranian University of Technology, PL-70322 Szczecin, Poland; emakuch@zut.edu.pl (E.M.); Paula.Ossowicz@zut.edu.pl (P.O.-R.); 4 Department of Microbiology and Environmental Chemistry, Faculty of Environmental Management and Agriculture, West Pomeranian University of Technology, PL-71434 Szczecin, Poland; Krystyna.Cybulska@zut.edu.pl; 5 Department of General and Transplantation Surgery, Pomeranian Medical University, PL-71252 Szczecin, Poland; sulikowskit@wp.pl

**Keywords:** fireweed, essential oil, antioxidant activity, antifungal activity, chemical composition, enhancement penetration, Franz diffusion cell

## Abstract

*Epilobium angustifolium* L. is a popular medicinal plant found in many regions of the world. This plant contains small amounts of essential oil whose composition and properties have not been extensively investigated. There are few reports in the literature on the antioxidant and antifungal properties of this essential oil and the possibility of applying it as a potential promoter of the skin penetration of drugs. The essential oil was obtained by distillation using a Clavenger type apparatus. The chemical composition was analyzed by the GC-MS method. The major active compounds of *E. angustifolium* L. essential oil (EOEa) were terpenes, including α-caryophyllene oxide, eucalyptol, β-linalool, camphor, (S)-carvone, and β-caryophyllene. The analyzed essential oil was also characterized by antioxidant activity amounting to 78% RSA (Radical Scavenging Activity). Antifungal activity against the strains *Aspergillus niger*, *A. ochraceus*, *A. parasiticum*, and *Penicillium cyclopium* was also determined. The largest inhibition zone was observed for strains from the *Aspergillus* group. The EOEa enhanced the percutaneous penetration of ibuprofen and lidocaine. After a 24 h test, the content of terpene in the skin and the acceptor fluid was examined. It has been shown that the main compounds contained in the essential oil do not penetrate through the skin, but accumulate in it. Additionally, FTIR-ATR analysis showed a disturbance of the *stratum corneum* (SC) lipids caused by the essential oil application. Due to its rich composition and high biological activity, EOEa may be a potential candidate to be applied, for example, in the pharmaceutical or cosmetic industries. Moreover, due to the reaction of the essential oil components with SC lipids, the EOEa could be an effective permeation enhancer of topically applied hydrophilic and lipophilic drugs.

## 1. Introduction

The genus Epilobium (*Oenotheraceae*) includes about 200 species worldwide [1], with fireweed (*Epilobium angustifolium* L.) being one of the more popular species. This plant has long been used as a medicinal plant [2] due to its antioxidant [3,4,5,6], anti-inflammatory [3,7], and antimicrobial properties [7,8,9,10]. It is especially used in the treatment of benign prostatic hyperplasia [11]. Traditionally, the infusion of the leaves of this plant is used for colds, headaches, and gastrointestinal disorders [11]. The *E. angustifolium* herb contains biologically active compounds, mainly flavonoids and polyphenolic acids [8,11,12]. 

This plant contains a small amount of essential oil, the composition and biological effects of which are still not fully understood. Previous investigations of this plant have focused mainly on the analysis of alcoholic or water extracts [4,8,13,14]. However, more detailed studies were performed for the essential oil obtained from another variety of *Epilobium*, such as *Epilobium parviflorum* [15]. Only Zhang et al. obtained the essential oil from *E. angustifolium*, and assessed merely its chemical composition and antimicrobial activity [16] while other properties of this oil are not known yet. Natural essential oils exhibit, among others, antimicrobial, antioxidant, and anti-inflammatory properties [17]. In recent years, they have become more and more popular as ingredients in cosmetic and dermatological preparations. One of the main reasons to apply essential oils is the possibility to enhance the skin penetration of drugs [18]. 

The transdermal delivery of many drugs promises many benefits over an oral administration, such as satisfactory bioavailability due to avoiding first-pass hepatic effect and longer action of a drug as well as reduced side effects, and improved therapy due to the maintenance of plasma levels [19]. However, the SC provides an effective barrier to the penetration of most actives. Its main task is to prevent excessive water loss and to protect humans against microorganisms, allergens, and chemicals [20,21]. The SC is a thin membrane consisting mainly of keratinized epidermal cells, and the main intercellular components are lipids [22]. In recent years, there has been a search for natural permeation enhancers of low toxicity while maintaining their enhancing activity [19]. Therefore, more and more often the essential oils are also added to increase the skin penetration rate of some drugs.

Furthermore, dermatological and cosmetic products containing mainly “natural” ingredients are perceived by users to be safer as compared to “synthetic” ingredients [3]. The essential oils generally can be regarded as safe, because they are rapidly metabolized, not accumulated in the organism and quickly excreted after application to the skin [18]. In previous studies it was found that essential oils increased the delivery to the skin, among others: vitamins [23], ibuprofen [18,21,24], 5-fluorouracil, indomethacin [25], and chlorhexidine digluconate [26]. 

The aim of the study was to evaluate the composition, antioxidant and antifungal properties as well as assessment of the enhancement of the skin penetration, of three model substances (ibuprofen, lidocaine, and caffeine) by *E. angustifolium* essential oil.

## 2. Results

### 2.1. Essential Oil with E. angustifolium L. (EOEa)

After steam distillation in Clavenger type apparatus from 1200 g of dry *E. angustifolium* herb, 0.523 g (0.035%) of the essential oil was obtained. The isolated essential oil was light yellow in color with a specific and strong odor (Figure S1). 

**Figure 1:**
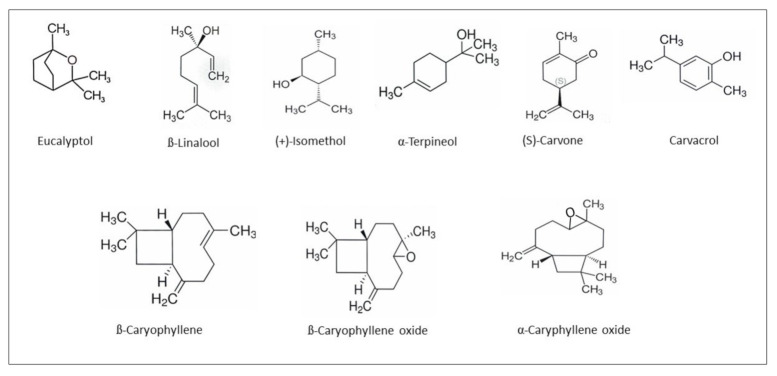
The structural formulas of the main terpenes identified in the *E. angustifolium* essential oil.

Table 1 presents the major components of *E. angustifolium* essential oil determined with gas chromatography-mass spectrometry (GC-MS). Qualitative identification makes it possible to identify 24 compounds accounting for a total of 99%. The highest concentration of cosanes (23.70%), 5-methyldocosane (14.95%) and α-caryophyllene oxide (8.57%) were found. Another large group was terpenes—their content ranged from 4.54% to 1.94%. Moreover, the analyzed essential oil contained 9.22% of caryophyllenes (α-caryophyllene and β-caryophyllene) and 11.30% oxygen derivatives of caryophyllenes (α-caryophyllene oxide and β-caryophyllene oxide). The following terpenes have also been identified: sesquiterpenes, such as δ-cadinene, γ-cadinene, β-cadinene, then germacradien-4-ol as well as monoterpenes, such as β-linalool, (S)-carvone, camphor, and eucalyptol and in smaller quantities (+)-isomenthol, α-terpineol, α-terpinyl acetate, thymol, and carvacrol—Table 1. Figure 1 shows the structural formulas of the main terpenes identified in the analyzed essential oil.

### 2.2. Antioxidant Activity 

Table 2 presents the antioxidant activity of EOEa, expressed in Trolox equivalent (mg Trolox/dg EOEa) and a percentage of free radical scavenging activity (% RSA). The analyzed essential oil exhibited high antioxidant properties, confirmed by the DPPH method. The antioxidant activity was 2.445 ± 0.025 mg Trolox/g EOEa, which corresponded to 78.021 ± 0.755% RSA.

### 2.3. Antifungal Activity

Two genera of fungi were used in the research, i.e., *Aspergillus* and *Penicillium*. The EOEa showed antifungal activity. However, it was dependent on the analyzed strain (Table 3). The zones of inhibition ranged from 7 mm to 28 mm on average, with much stronger reactions observed in the case of *Aspergillus* (Figure S2). 

### 2.4. In Vitro Skin Penetration Studies

The penetration tests were carried out using emulsions containing the analyzed drugs as well as the EOEa. After preparing the emulsions, their stability and separation was tested. Both tests for all emulsions showed good physical properties. All emulsions were uniform in color and showed no separation (Figure S3). 

**Figure 2:**
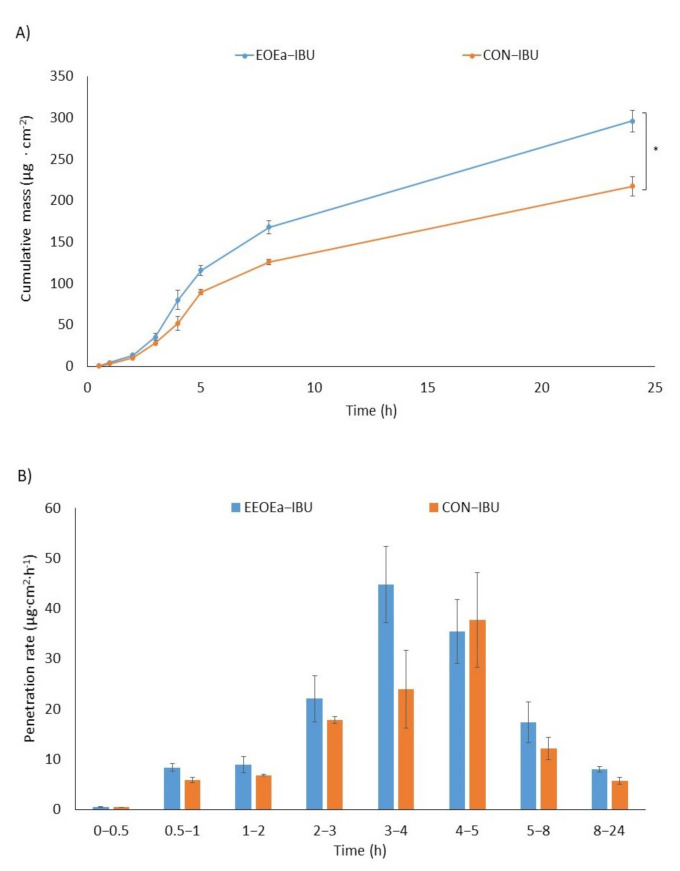
The cumulative mass of ibuprofen in the acceptor fluid (**A**) and the penetration rate (**B**) of ibuprofen through the skin during the 24 h experiment, EOEa-IBU—emulsion containing the *E. angustifolium* essential oil, CON-IBU—control—an emulsion without the *E. angustifolium* essential oil. Each point and bar represents the mean ± standard deviation SD (*n* = 3). For * *p* < 0.0001 versus the control.

**Figure 3:**
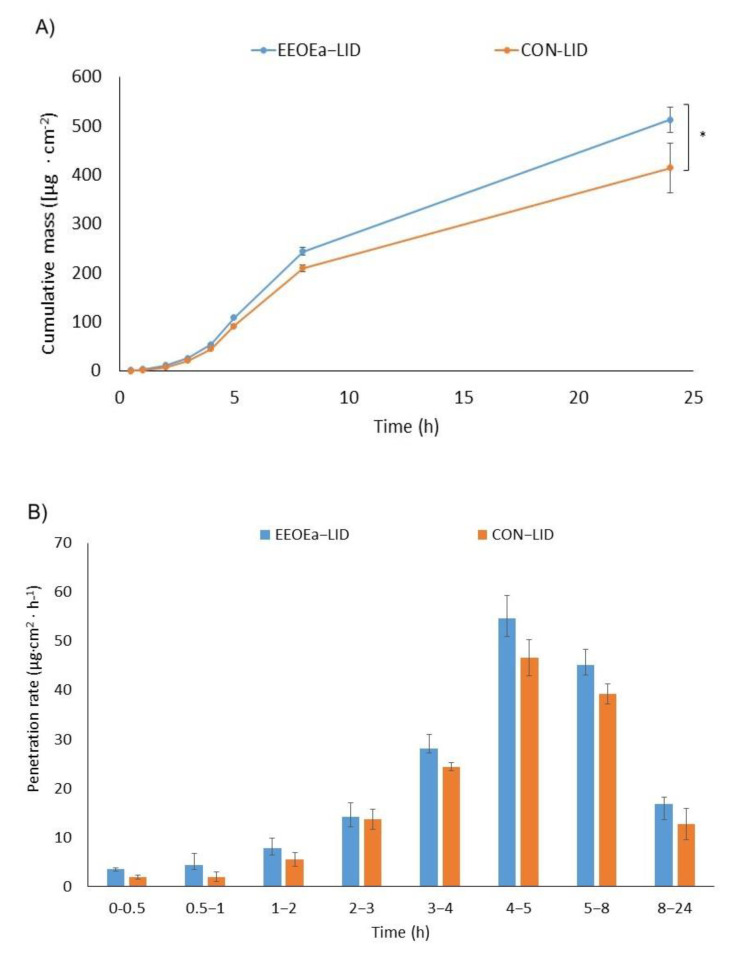
The cumulative mass of lidocaine in the acceptor fluid (**A**) and the penetration rate (**B**) of lidocaine through the skin during the 24 h experiment, EOEa-LID—emulsion containing the *E. angustifolium* essential oil, CON-LID—control—an emulsion without the *E. angustifolium* essential oil. Each point and bar represents the mean ± standard deviation SD (*n* = 3). For * *p* < 0.0001 versus the control.

**Figure 4:**
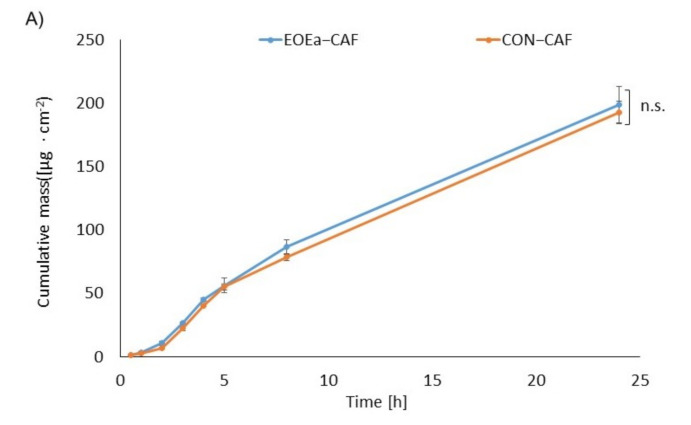

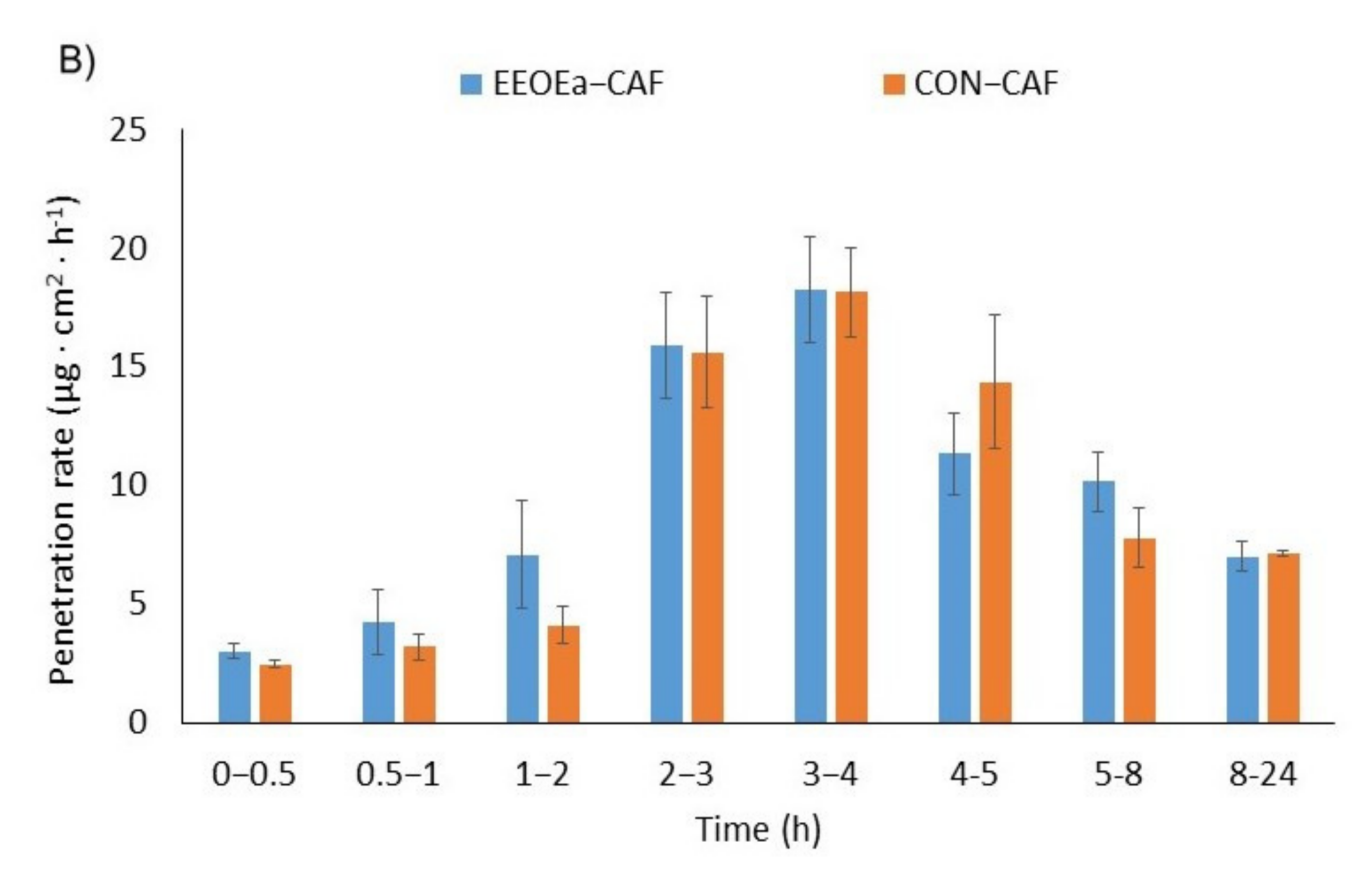
The cumulative mass of caffeine in the acceptor fluid (**A**) and the penetration rate (**B**) of caffeine through the skin during the 24-h experiment, EOEa-CAF—emulsion containing the *E. angustifolium* essential oil, CON-CAF—control—an emulsion without the *E. angustifolium* essential oil. Each point and bar represents the mean ± standard deviation SD (n = 3). n.s.—no significant differences.

The effect of EOEa on the in vitro skin permeation of ibuprofen, lidocaine, and caffeine through pig skin is shown in Table 4. Figure 2, Figure 3 and Figure 4 show the cumulative mass and the penetration rate determined during the entire test period. The use of EOEa increased the flux (Jss) in the case of ibuprofen and lidocaine, while a slight decrease was observed in the case of caffeine. The transdermal flux of ibuprofen with the use of EOEa was more than 1.3 times higher than that without (35.162 and 26.238 µg/cm^2^∙h, respectively). The permeability coefficient (K_P_) was also about 1.3 times higher in the case of ibuprofen using EOEa. A similar effect was observed with lidocaine. The transdermal flux of this drug with the use of essential oil was about 1.2 times higher than without oil (41.439 and 35.578 µg/cm^2^∙h, respectively). K_P_ value was also 1.2 times higher in the case of lidocaine using EOEa. In the case of caffeine, only slight differences were observed. Another parameter determined was the lag time (L_T_). No significant effect of the use of the EOEa on this parameter in the case of ibuprofen and lidocaine was found. In contrast, after caffeine application, the L_T_ was slightly decreased by introducing the EOEa. The diffusion coefficient (D) in the skin was similar to the ibuprofen and lidocaine regardless of whether it was applied with or without the EOEa. However, there was a difference for caffeine—the diffusion coefficient was higher if the oil was used and it was 3.302 cm^2^/h, in contrary to 2.652 cm^2^/h without EOEa. The ability of the drug to release from the vehicle and transport into the outermost layers of the stratum corneum (K_m_) ranged from 0.304 to 0.230 for caffeine without and with the use of EOEa, from 0.565 to 0.722 for ibuprofen without and with the use of EOEa, and from 1.039 to 1.185 for lidocaine without and with the use of essential oil, respectively.

The cumulative mass, determined after 24 h of the study, was significantly higher after the adding of EOEa to the vehicle in the case of ibuprofen and lidocaine (*p* < 0.0001)—Figure 2, Figure 3 and Figure 4. The sudden increase in penetration was observed between 3 and 4 h for the ibuprofen and caffeine, while in the case of lidocaine between 4 and 5 h. The addition of EOEa to the emulsion resulted in faster and more efficient penetration of ibuprofen, compared to the control (Figure 2b).

### 2.5. The Effect of EOEa on the Penetration of Studied Compounds

In order to more precisely elucidate the effects of terpenes contained in the essential oil on the penetration of analyzed drugs, it was decided to expand the analysis of penetration of active substances contained in the EOEa as well as their accumulation in the skin after 24 h of study. In addition, the FTIR method was used to determine the effect of EOEa on the skin lipids.

#### 2.5.1. GC-MS of Skin and Acceptor Fluid after 24-h Penetration

**Figure 5:**
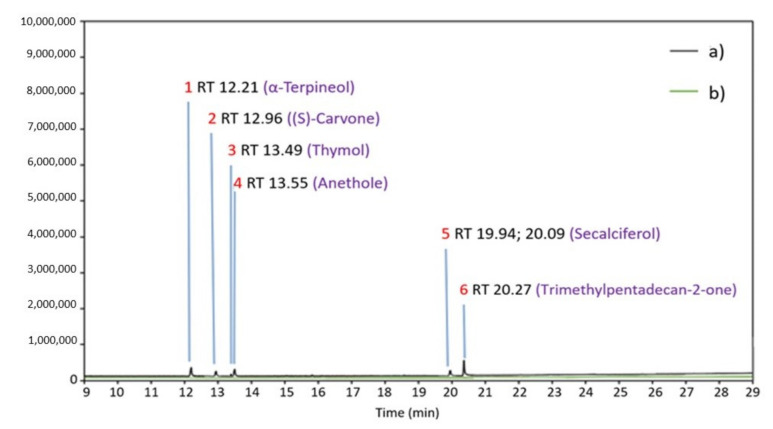
GC-MS chromatogram of the samples after skin extraction after 24 h of penetration of emulsion contained the EOEa (**a**, black line) and of the acceptor fluid after 24 h of penetration of emulsion contained the EOEa (**b**, green line), RT—retention time (min).

Figure 5 shows the main components determined with gas chromatography-mass spectrometry (GC-MS) occurring in skin samples taken after 24 h of penetration as well as in the acceptor fluid collected after the end of the study. The GC-MS analysis showed that the following compounds have accumulated in the skin: α-terpineol, (*S*)-carvone, thymol, anethole, secalciferol, trimethylpentadecan-2-one. However, no active compounds present in EOEa were found in the acceptor fluid collected at the end of the study (Figure 5).

#### 2.5.2. The FTIR Spectra of Skin before and after EOEa Application

**Figure 6:**
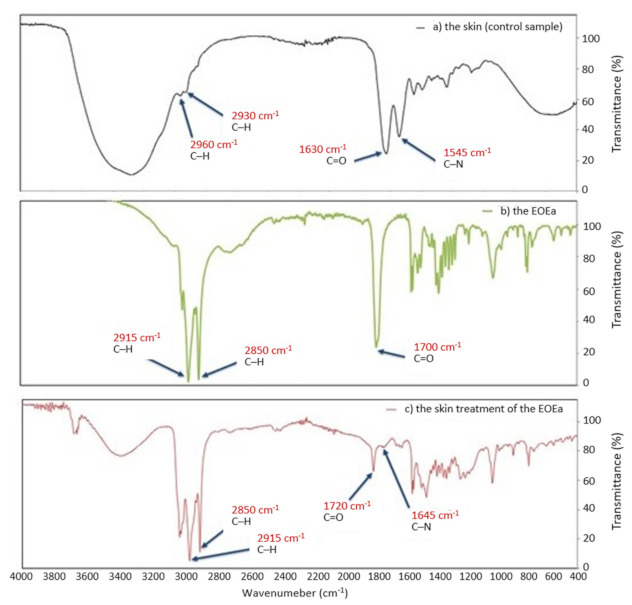
FTIR spectra of pig skin before and after 24 h treatment: (**a**) control sample, (**b**) *E. angustifolium* essential oil (EOEa), (**c**) the skin treated with *E. angustifolium* essential oil (EOEa).

In Figure 6 FTIR spectra of pig skin before and after 24 h treatment with the EOEa are presented. The lipid extraction resulting from the EOEa treatment of the skin was evaluated by comparing the intensities of the C–H bonds after the skin treatment of EOEa (Figure 6c) to the corresponding peaks with EOEa (Figure 6b) and control sample—skin before application of EOEa (Figure 6a). The FTIR spectrum of the control sample (Figure 6a) shows small peaks of the C–H of the alkyl groups (2960 and 2930 cm^−1^). The FTIR spectrum of the *E. angustifolium* ethanol extract containing the essential oil, was discussed in more detail in our previous publication [8]. 

## 3. Discussion

In recent years, essential oils have been used as new alternatives in the production of cosmetics and pharmaceuticals mainly due to their antioxidant and antimicrobial properties [27]. In addition, due to the high content of terpenes, they are used more and more often as promoters of the penetration enhancers of active substances through the skin [18,21,24]. There are many aromatic plants around the world showing such an effect. However, increased interest has been focused on searching for interesting natural sources, rich in valuable essential oils. Such a plant is *E. angustifolium*, which is characterized by a small amount of essential oil. However, due to its rich composition and biological activity it may be an interesting candidate for use in various industries. In our study, steam distillation was applied to obtain the essential oil from *E. angustifolium.* It was found that has this oil demonstrated antifungal, antioxidant activity and can also be a potential promoter of drug penetration through the skin. In addition, some substances contained in essential oils penetrate deeply into the skin and accumulate there so there may be beneficial due to their biological effect.

### 3.1. Isolation and Composition of EOEa 

In the process of steam distillation from 1200 g of dry *E. angustifolium* herb, 0.523 g (0.035% (*w*/*w*)) of the essential oil was obtained. A slightly lower yield of *E. parviflorum* essential oil, amounting to 0.023% (*w*/*w*), was obtained by Bajer et al. [15], while higher with *Epilobium hirtusum* (0.8% (*w*/*w*)) was obtained by Eghmazi et al. [28]. The essential oil obtained in our study was yellow in color, similar to the essential oil obtained from *E. hirtusum* by Bajor et al. [15]. In our study, the EOEa chemical composition was determined by the GC-MS analysis. A large group were the compounds from the terpenes and terpenoids group and cosanes and methyldocosane. The analyzed essential oil contained caryophyllenes (i.e., α-caryophyllene and β-caryophyllene) and their oxygen derivatives (i.e., α- and β-caryophyllene oxide). The presence of caryophyllenes in the essential oil obtained from *E. angustifolium* is also confirmed by Zeng et al. [16]. The occurrence of these terpenes and terpenoids (α-caryophyllene, β-caryophyllene, and α- and β-caryophyllene oxide) was also detected by others in essential oils obtained from other *Epilobium* species, such as *E. parviflorum* [15] and *E. hirtusum* [28]. Moreover, these compounds were also identified in water-methanol extracts (25/75) obtained from both fresh and dried plant material [29]. The caryophyllenes and their derivatives are frequently isolated components of essential oils [30], responsible for their unpleasant aroma [31]. The caryophyllene is a natural bicyclic sesquiterpene usually found in many essential oils, among others clove, rosemary. This compound is mainly used as a flavoring or fragrance enhancer in spice blends, citrus flavors, soap, detergents, creams or lotions, food products, and beverages [32]. Adamczak et al. suggest that the caryophyllenes are one of the more numerous groups of compounds found in the essential oil of *E. angustifolium* [11]. In our research, other compounds belonging to the terpenes group were also identified: sesquiterpenes, such as δ-cadinene, γ-cadinene, β-cadinene, then germacradien-4-ol and monoterpenes, such as β-linalool, (S)-carvone, camphor, and eucalyptol as well as in a smaller amount: (+)-isomenthol, α-terpineol, α-terpinyl acetate, thymol, and carvacrol. Similarly, the sesquiterpenes in *E. hirtusum* essential oil were found by Zeng et al. [16] who identified: α-cadinol and germacrene D, while among monoterpenes: α-terpinene, γ-terpinene, and terpinol. Bajer et al. [15] confirmed the presence of monoterpenes: linalool as well as (Z)-linalool furanoxide, (E)-linalool furanoxid, camphor, 4-tepineol and α-terpineol, carvon and thymol in the essential oil of *E. partiflorum*. They also identified (E)-anethole, which was also detected in our research. In the essential oil analyzed in our study, the presence of higher alkanes contained more than 10 carbon atoms, namely cosanes and the methyl derivative (5-methyldocosane), was shown. The content of components with a similar structure in the essential oil of *E. angustifolium*, such as tricosane, tetracosane, pentacosane, Z-14-nanocosane, was confirmed by Zeng et al. [16]. Eicosane, docosane, and tricosane were identified in the essential oil of *E. hirsutum* [15]. Some components identified in our study were also found by Kaskoniene et al. in water-methanol extracts (75% *v*/*v*) from fresh and dried herb of *E. angustifolium*. They were *cis-trans*-anethole, γ-terpinen-7-al., α- and β-caryophyllene, γ- and δ-cadinene [29]. A slightly different composition of essential oils from the *Epilobium* group observed by other authors could probably depend on the geographical origin, plant chemotype, vegetation phase and the part of the plant from which the oil was obtained [33,34,35]. In addition, volatile compounds contained in essential oils are very susceptible. Therefore the harvesting method, transport, storage, and packaging play a particularly important role [15,36]. 

### 3.2. Antioxidant Activity of EOEa

The aromatic plants synthesize many secondary metabolites that possess many pharmacological properties and other antioxidant activities [37]. The high antioxidant activity is very desirable, especially in cosmetic and dermatologic preparations, because antioxidants play a vital role in skin regeneration and prevent losing elasticity and inhibit the aging process [3,38]. Many factors can disturb the oxidative balance, leading to oxidative stress. The oxidative stress leads to the increase and consequent attack of reactive oxygen species (ROS), such as alkoxyl (RO•), superoxide anion (O_2_•), hydroxyl (HO•), and peroxyl (RO_2_•) radicals [39]. Therefore, it is crucial to support the body’s endogenous protective system with exogenous antioxidants because the skin is constantly exposed to harmful external factors that contribute to the increased formation of free radicals [40]. The antioxidant activity of plants is mainly due to the presence of polyphenolic compounds. However, it turns out that the terpenes, which are components of essential oils, also show a similar effect [41,42]. In our study, the EOEa was characterized by antioxidant activity. There are a very few reports in the literature on the antioxidant activity of the essential oil of the genus *Epilobium*. Previous papers only contain information on the antioxidant activity of ethanol extracts [4,8,9,38,43], methanol extracts [4,29], isopropanol extracts [4], and water extracts from *E. angustifolium* [4,9,44]. The essential oils consist of different organic compounds that contain conjugated carbon double bonds and hydroxyl groups responsible for inhibiting free radicals [39]. In our study, the EOEa contained a significant amount of caryophyllenes (i.e., α- and β-caryophyllene, caryophyllene oxide) belonging to the group of sesquiterpenes, which could be responsible for the antioxidant effect [37,38]. Some monoterpene hydrocarbons found in EOEa such as α- and γ-terpinene, also showed high antioxidant activity [35]. Other studies have demonstrated the antioxidant activity of carvacrol, thymol, and *p*-cymene [42,45] as well as linalool, eucalyptol, and α-terpineol [46,47]. 

### 3.3. Antifungal Activity of EOEa 

The use of essential oils as antifungal agents has been known for a very long time, both in the treatment of fungal infections [48,49] as well as in the food industry as natural antifungal agents. Generally recognized as safe, the natural essential oils possess a broad spectrum of fungicidal activity [50]. In recent years, plants that could be a new source of such valuable substances have attracted more interest. Therefore, in our study, we initially estimated the fungistatic effect of EOEa. As model fungi, two common mold species, such as *Aspergillus* and *Penicillium*, were selected to evaluate the zones of inhibition of their growth by EOEa. The analyzed essential oil showed antifungal activity, especially against strains of *Aspergillus*. The fungi of the genus *Aspergillus* are ubiquitous filamentous fungi found all over the world. To date, over 185 *Aspergillus* species have been identified, 20 of which have been reported to cause harmful infections in humans, animals as well as plants [51]. The *A. flavus* is one of the more dangerous fungal species, due to its interference with direct infections in immunosuppressed patients [49,52]. The aflatoxins secreted by this strain are the most potent naturally occurring toxic and hepatocarcinogenic compounds, classified as group 1 human carcinogen by the International Agency for Research on Cancer [49]. There is no information about the antifungal activity of *E. angustifolium* oil in the available literature. Zeng et al. only describe the antibacterial activity against strain *Micrococcus luteus*, *Escherichia coli*, *Bacillus subtilis*, and *Enterobacter aerogenes* [16]. The essential oils obtained from other varieties of *Epilobium* also showed antimicrobial activity, for instance as *E. parviflorum* against the strains *Staphylococcus aureus*, *Enterococcus faecalis*, *Escherichia coli*, *Pseudomonas aeruginosa*, and *Candida albicans* [15] as well as *E. hirsutum* against the strains *Pseudomonas aeruginosa*, *Enterococcus faecalis*, *Mycobacterium smegmatis*, *Yersinia pseudotuberculosis*, *Staphylococcus aureus*, *Bacillus cereus*, *Salmonella enterica*, and *Escherichia coli* [28,53]. The antifungal activity of EOEa is mainly attributed to terpenes, which interfere with the structure of the fungal cell membrane [54]. Any disturbances in the synthesis of the cell membrane of the fungi result in damage and consequently death [54,55]. 

### 3.4. Skin Penetration 

The main barrier limiting the penetration of active substances through the skin is the SC, which prevents the entry of microorganisms, chemical substances and/or allergens to the body, as well as protects the organism against excessive water loss [20,21,56]. The SC is a thin membrane consisting primarily of cornified epidermal cells, while the main intercellular components are lipids [22]. In recent years, to increase skin penetration, many researchers have focused on a search for natural permeation enhancers with low toxicity [19]. Such substances could be found in essential oils, commonly regarded as valuable penetration promoters with additional antioxidant, antimicrobial, anti-inflammatory, and fragrance activity [10,57,58,59]. Such properties are also shown by *E. angustifolium* essential oil. However, it is very difficult to find information on its use as a promoter of the penetration of active substances through the skin in the available literature. Therefore, we decided to the assess the penetration of ibuprofen, lidocaine, and caffeine from emulsion with or without EOEa. Ibuprofen, lidocaine hydrochloride and caffeine were selected as they have been frequently included in topically applied pharmaceutical and cosmetic preparations. Moreover, these drugs are often used as model drugs in in vitro penetration studies. They are characterized by different activities and belong to different therapeutic groups. Ibuprofen is a very popular non-steroidal analgesic and anti-inflammatory drug [20]. The lidocaine is used as topical anesthetic in dermatology and cosmetology to alleviate the pain from non-surgical cosmetic procedures [60]. Caffeine is very popular anti-aging agent applied in cosmetic preparations. [61]. The lipophilicity of caffeine is log *P* = −0.07 [62], for lidocaine hydrochloride log *P* ≤ 0 [63] and for ibuprofen, log *P* is in the range of 2.41–4.00 [64]. 

In our study, the penetration test was carried out using a Franz diffusion cell. The acceptor fluid was PBS (pH 7.4), while the emulsion containing the active substances with and without the EOEa was placed in the donor chamber. The penetration test was performed using the pig skin. The porcine skin is frequently used for the evaluation of percutaneous permeation of topically applied active substances. Numerous histopathological studies confirmed its similarity to human skin. The SC of human and pig skins is very similar and consists of dense flatted epithelial cells. Moreover, the thickness of the layers, i.e., stratum basale, spinosum, and granulosum are comparable [65,66]. In our study, the parameters of skin permeability were determined to compare the effect of EOEa on the penetration of various drugs (such as caffeine, lidocaine, and ibuprofen). The obtained results suggest that the EOEa used can promote repartition in the skin of some drugs. The use of EOEa significantly increases the flux in the case of ibuprofen and lidocaine. The cumulative mass of these drugs, determined after 24 h of the study, was significantly higher after adding EOEa to the emulsion. Similarly, enhancement of ibuprofen penetration has been demonstrated by others as a result of using clove oil, angelica oil, chuanxiong oil, cyperus oil, and cinnamon oil [18,21,24]. Moreover, the terpenes used alone, such as anethole, menthone, and eugenol, significantly enhanced valsartan penetration through the rats’ skin [19]. Also menthol and cineole increased the penetration of lidocaine from the composite patches [67]. The terpenes contained in the essential oils can increase skin permeation of drugs primarily through interaction with SC lipids and by increasing the solubility of the drug into SC lipids [68,69] which was confirmed in our study (see Section 3.4.2). In the case of lipophilic drugs, terpenes increase the stratum corneum/vehicle partition coefficient. The penetration of lipophilic drugs increases in proportion to their solubility in the enhancer (or enhancer solution). In contrast, the penetration of hydrophilic drugs is improved due to an increased diffusion coefficient [69]. In our study the highest penetration as compared to control was observed for ibuprofen, the most lipophilic compared with lidocaine hydrochloride and caffeine. The most negligible difference in the cumulative mass after 24 h penetration of formulations with or without EOEa was observed for caffeine, a typically hydrophilic compound.

#### 3.4.1. The Penetration of Active Compounds of EOEa and Their Accumulation in the Skin—GC-MS Analysis

In our study, terpenes were the main components of the analyzed essential oil. Due to their well-known biological activity, the analysis of their accumulation in the skin is an important factor assessing their enhancing effects on the penetration of active substances. Penetration enhancers, with high biological activity, should not or only in negligible amounts penetrate through the skin. Therefore, it is also essential to analyze the penetration of these substances into the acceptor fluid [69]. Cal et al. showed that these substances primarily accumulated in the skin, while their penetration into the skin depends mainly on the vehicle used and log P of terpenes [59]. Our study demonstrated that some of the terpenes contained in the essential oil, i.e., α-terpineol, (S)-carvone, thymol, and anethole, accumulated in the skin, while the concentrations of these compounds in acceptor fluid collected after 24 h study were not determinable. The accumulation of terpenes in the skin may be beneficial, due to their effect on skin lipids (see Section 3.4.1.) and their antioxidant, antimicrobial, anti-inflammatory, or analgesic activity [69,70,71,72]. 

#### 3.4.2. FTIR of Skins after EOEa Application on the Skin

As previously mentioned, one of the main groups of compounds responsible for enhancing skin penetration of certain active substances are terpenes. Their action is mainly based on changing the structure of the SC barrier and interaction with intercellular lipids [18]. Instrumental methods can determine the effect of the terpenes on skin lipids, among others, Fourier transformed infrared spectroscopy (FTIR). FTIR has already been proved to be a promising tool to study the spatial organization of *stratum corneum* lipids [18]. Therefore, in our study, we decided to perform an FTIR analysis after topical application EOEa on the skin surface. The study on the effect of the essential oil on skin cells was carried out under the same conditions as above, using a Franz diffusion cell for 24 h. In our study, the resultant lipid extraction was evaluated by comparing the intensities of the C–H bonds after the skin treatment with EOEa as compared with skin without essential oil. The FTIR spectrum of the control sample shows small peaks C–H of the alkyl groups (2960 and 2930 cm^−1^), which are attributable to the long alkyl chains of ceramides, cholesterol, and fatty acids i.e., the major components of the SC lipids [19], while the FTIR spectrum of the treated skin with EOEa shows intense peaks of the C–H of the alkyl groups [73]. The difference between the intensity of C–H bonds of the control sample and the skin treated with EOEa treated was pronounced. The results revealed that the EOEa promoted the skin permeation of some active substances mainly by disturbing rather than extracting the SC lipids [19]. The strong band at 1630 cm^−1^ is connected to the C=O stretching vibration and at 1545 cm^−1^ arises from the C-N banding vibration, of SC proteins. The FTIR spectrum of skin treated with essential oil (Figure 6c) shows a shift of the peaks C=O stretching vibration and C-N banding vibration. The SC is composed of lipids (ceramides) that are tightly packed as bilayers due to a high degree of hydrogen bonding. It is likely that the penetration of the essential oil terpenes into the lipid bilayers of SC leads to the disruption of the hydrogen bond network at the head of ceramides [74]. Other authors obtained similar results. For example, Ahad et al. evaluated the lipids after skin extraction resulting from the treatment of anethol. The FTIR spectra of SC treated with terpenes exhibited decreased height and area of asymmetric and symmetric C–H [19]. The main barrier in transdermal drug delivery is the lipophilic part of SC in which lipids (mainly ceramides) are tightly connected, which imparts barrier property of SC. However, if the skin is treated with terpenes, ceramides may be loosened [19]. The disruption of the highly ordered structure of SC lipids with increased intercellular diffusivity could support the penetration of active agents [75]. In addition, our studies confirmed that the essential oil is absorbed into the skin (Figure 6c). The FTIR spectrum of skin treated with the EOEa shows the same penetration bands as observed in EOEa.

## 4. Materials and Methods

### 4.1. Chemicals 

2,2-diphenyl-1-picrylhydrazyl (DPPH), 6-hydroxy-2,5,7,8-tetramethylchroman-2-carboxylic acid (Trolox), ibuprofen, lidocaine hydrochloride, and caffeine were purchased from Sigma Aldrich (Poznań, Poland); disodium phosphate and potassium dihydrogen phosphate from Merck, Darmstadt (Germany); Biobase, beeswax were purchased from Mazidła.com (Poznań, Poland); grape seed oil was purchased from Monini (Spoleto, Italy); sodium acetate anhydrous, potassium persulfate, potassium acetate, xylene, 99.5% acetic acid, aluminium chloride, 36% hydrochloric acid, sodium chloride, potassium chloride, sodium sulphate anhydrous as well as propylene glycol, ethanol, and methanol were from Chempur (Piekary Śląskie, Poland), whereas acetonitrile for HPLC from J.T. Baker, (Landsmeer, The Netherlands). All reagents were of analytical grade. 

### 4.2. Plant Materials 

The plant raw material consisted of dried herbs from *E. angustifolium* and was purchased from a local certificated herbal store (Nanga, Złotów, Poland). The first portion of the plant material was applied to the steam distillation to obtain the essential oil. In contrast, the second portion was divided into the samples (100 g each) and deposited (nr: EA-AM2020-09) in the Chair and Department of Cosmetic and Pharmaceutical Chemistry of the plant material warehouse Pomeranian Medical University. 

### 4.3. Distillation of Essential Oil

The steam distillation of the essential oils was carried out using a Clavenger type apparatus for 6 h until the last portion of the essential oil was obtained. A total of 12 series were carried out, each with 100 g of dry plant material and 1000 mL of distilled water. The essential oil was collected after each series, and finally all portions were combined into one collective sample. Next, the essential oil was dried over anhydrous sodium sulfate and stored at 4 °C, in a sealed dark vial until analysis. 

### 4.4. Antioxidant Activity EOEa 

The stable DPPH radical scavenging activity was measured as previously described with minor modification [8,33,37]. Shortly, an aliquot of 150 µL of the studied samples was mixed with 2850 µL of 0.3 mM DPPH radical solution dissolved in methanol. After 10 min of incubation in the dark at room temperature, absorbance measurement at 517 nm was performed using Hitachi UV-Vis Spectrophotometer U-5100. Three independent samples of each examined extract were prepared. As a reference, 6-hydroxy-2,5,7,8-tetramethylchroman-2-carboxylic acid (Trolox) was applied. The results are presented as Trolox equivalents in mg Trolox/g EOEa. and as an inhibition percentage (%RSA—radical scavenging activity) using the following equation:

$$\%\mathrm{RSA}=\left(1-\frac{\mathrm{As}}{\mathrm{Ac}}\right)\;\times \;100\%$$

where: A_s_—absorbance of the tested sample; A_c_—absorbance of the control sample. 

### 4.5. Antifungal Activity 

The following microbial strains were used in the studies: *Aspergillus niger*, *Aspergillus ochraceus*, *Aspergillus parasiticus*, *Penicillium cyclopium*. The sensitivity of test microorganisms to EOEa was determined by the method of diffusion into the agar medium, using the well variant [76]. PDA (Potato Dextrose Agar) medium was used to cultivate the fungi. The appropriate medium (20 mL) was poured into Petri dishes with a diameter of 90 mm, and after solidification of the medium, 5 wells with a diameter of 4 mm were cut out using a sterile cork borer. Onto these prepared Petri dishes, a fungi inoculum was introduced, consisting of a spore suspension in physiological saline supplemented with 0.1% peptone and 0.25% Tween 20. The plates with the sown strains were allowed to completely absorb the fluid for about 60 min, and then 10 µL of previously prepared samples of EOEa at the concentrations of 12.5%, 25%, 50%, and 100% were introduced into 4 wells. 10 µL of sterile water was introduced into the well in the center of the dish as a control. The fungi cultures were incubated at 25 °C for 7 days. The inhibitory effect of the test substances was assessed based on the inhibition zone of the growth of the culture. 

### 4.6. In Vitro Skin Permeation Studies 

In the in vitro penetration experiments, an abdomen porcine skin was used due to their similar permeability to human skin. Numerous histopathological studies confirmed its similarity to human skin [60,61]. The porcine skin obtained from the local slaughterhouse was used. The fresh abdominal porcine skin was washed in PBS buffer pH 7.4 several times. The skin of 0.5 mm in thickness was dermatomed. Thereafter, it was divided into 2 cm × 2 cm pieces. The skin samples were wrapped in aluminum foil and stored in a freezer at −20 °C until use, not longer than three months. This frozen storage time was safe to keep skin barrier properties [73]. The permeation experiments were performed in the Franz diffusion cells (SES GmbH Analyse Systeme, Bechenheim, Germany) with a diffusion area of 1 cm^2^. The acceptor chamber was filled with PBS solution (pH 7.4). In each diffusion unit, a constant temperature of 32.0 ± 0.5 °C [74] was maintained via a thermostat (VEB MLW Prüfgeräte-Werk type 3280, Leipzig, Germany). The acceptor chamber content was stirred with a magnetic stirring bar at the same speed for all cells. The donor chamber volume was 2 mL, and the volume of the acceptor chamber was 8 mL. On the day of the experiment, the skin samples were slowly thawed at room temperature for 30 min and were hydrated by PBS pH 7.4 [61,62,63]. Undamaged pieces of skin were placed in the Franz diffusion cell between donor and acceptor chamber. The integrity of the skin was measured. For this purpose, an LCR meter 4080 (Voltcraft LCR 4080, Conrad Electronic, Landenweg, Germany), operated in parallel mode at an alternating frequency of 120 Hz (error at kΩ values <0.5%), was used. The tips of measuring probes were immersed in the donor and acceptor chamber, filled with PBS (pH 7.4) as described previously [75,76]. Only skin samples with impedance >3 kΩ were used. These values are similar to the electrical resistance of human skin [76]. Thereafter, a defined dose (1 g) of each emulsion was applied to the skin’s outer side in donor compartment. All donor chambers were closed with plastic stoppers. The penetration study was carried for 24 h. At the time points of 1, 2, 3, 5, 8, and 24 h, 0.5 mL of acceptor samples were withdrawn and the chamber was refilled with the same volume of a fresh portion of PBS pH 7.4. The ibuprofen, lidocaine, and caffeine concentrations in the acceptor phase were measured by the HPLC method. The cumulative mass (µg) of each drug studied was calculated based on the obtained concentration. After the end of the experiment, diffusion cells were disassembled, and the skin samples were analyzed for the content of active substances found in the EOEa. The accumulation of active substances in the skin after penetration was determined using a modification of the method described by Haq et al. and Ossowicz-Rupniewska et al. [64,77]. After 24 h of the experiment, each skin sample was removed. In order to remove vehicle residues, they were carefully rinsed with 0.5% sodium lauryl sulfate solution. The skin was then cut around the diffusion area (1 cm^2^) and dried at room temperature. Each of 1 cm^2^ skin samples was cut into small pieces, placed in 2 mL methanol, and incubated for 24 h at 4°C. After this time, skin samples were homogenized for 3 min using a homogenizer (IKA^®^T18 digital ULTRA TURRAX, Staufen, Germany). The homogenate was centrifuged at 3500 rpm (1712× *g*). The supernatant was collected for GC-MS analyses with pure methanol applied as a control. Based on the drug permeation profiles, permeation parameters such as the steady-state permeation flux (Jss), the diffusion coefficient (KP), and the time required to reach steady-state permeation (lag time—LT) were determined and compared depending on the type of vehicles and the concentration of drugs in studied preparation. The steady-state fluxes (JSS) of drugs through the skin were calculated from the slope of the plot of cumulative mass in the acceptor phase over time and were expressed as the amount of compound per skin area and time (μg·cm^−2^·h^−1^). Lag time was determined by extrapolating the equation and KP is a quantitative measure of the rate at which a molecule can cross a skin barrier. 

### 4.7. Preparation of the Emulsion

The emulsion (W/O) was prepared according to a modified method by Suñer-Carbó et al. and Nowak et al. [3,78]. Sequentially, the emulsion was prepared by slow addition of the oil phase to the aqueous phase at a temperature of 80 °C under continuous stirring at 250 rpm. The oil phase consisted of lipophilic components such as Biobase, grape seed oil, and beeswax. Biobase is a commercial preparation containing glyceryl stearate, cetearyl alcohol, and sodium stearoyl lactylate (Mazidła.com, Poznań, Poland). The water phase consisted of propylene glycol and water. The resulting mixture was stirred until a homogeneous emulsion was completely formed. The essential oil was added after vehicles cooled down. Then the individual drugs were weighed and dissolved: ibuprofen was dissolved in a small volume of ethanol (400 µL), while lidocaine hydrochloride and caffeine in a small volume of water (400 µL) and mixed with the vehicle. The final emulsion was transferred to the package and stored in the dark at room temperature. The studies were carried out in triplicate for each vehicle. Three emulsions containing EOEa and ibuprofen (EOEa–IBU), lidocaine (EOEa–LID), or caffeine (EOEa–CAF) were obtained. Similarly, as control, emulsions were prepared without the essential oil (CON–IBU, CON–LID, and CON–CAF, respectively for ibuprofen, lidocaine, and caffeine). Composition of the emulsions prepared are provided in Table 5.

### 4.8. Stability of Preparations

The stability of all emulsions was tested. This parameter was evaluated by centrifuge test. The separation of the emulsions was evaluated by centrifuge test. The emulsions samples (3 g) were centrifuged (MPW-223e, Mechanika Precyzyjna, Warsaw, Poland) at 4000 rpm at 25 °C for 10 min to establish the possibility of emulsion breaking. Moreover, the stability of all emulsions was also evaluated using the heating-cooling test: incubation at 45 °C (Drying Oven, DHG-9075A) for 48 h, followed by incubation at 4 °C (in refrigerator) for 48 h. 

### 4.9. GC-MS Analysis 

The qualitative and quantitative composition of EOEa components and analyzed ingredients in the liquid after skin extraction and in the acceptor fluid were analyzed by gas chromatography-mass spectrometry (GC-MS). Chromatographic analyses were performed with a TRACE GC series apparatus with a VOYAGER mass detector using a DB5 capillary column (30 m × 0.25 μm × 0.5 μm). The following separation parameters were used for the analysis: helium flow of 1.0 mL/min, sample chamber temperature of 240 °C, and detector voltage of 350 V. The thermostat temperature increased according to the following program: isothermal at 50 °C for 1 min, increase at a rate of 8 °C/min, isothermal at 260 °C for 5 min, and then cooled to 50 °C. The sample partition coefficient in the dispenser was 20, the volume of dispensed sample was 1 µL, and the ion mass range was 25–350 mV/z. The quantitative composition of individual compounds was determined assuming that the sum of all identified compounds is 99%.

### 4.10. FTIR-ATR Analysis

The pig skin samples were analyzed by FTIR-ATR technique according to the modified method of Ahad et al., Chen et al. [16,66]. The skin was prepared according to the procedure described in Section 4.6. The 0.1 g EOEa was applied to the skin, the study was conducted for 24 h (equivalent to the duration of permeation studies). After the test, each skin disc was washed, blotted dry, and air-dried for 2 h. The pure skin, without essential oil, was the control. The functional groups occurring in the tested skin samples were analyzed using the Fourier transformed infrared (FTIR) spectra. The spectra were obtained using Thermo Scientific Nicolet 380 spectrometer (Thermo Fisher Scientific, Waltham, MA, USA) equipped with an ATR diamond plate. Thirty-two scans were acquired in the 4000–400 cm^−1^ range.

### 4.11. HPLC Analyses

The concentration of drugs in acceptor fluid was determined by high-performance liquid chromatography (HPLC), using the HPLC system from Knauer (Berlin, Germany). The tested components were separated on a 125 mm × 4 mm column containing Hyperisil ODS (C18), particle size 5 µm. The column temperature was set at 25 °C, injection volume was 20 µL. The mobile for ibuprofen consisted of 0.02 mol/dm^3^ potassium dihydrogen phosphate-acetonitrile-methanol (45/45/10, *v*/*v*/*v*); for lidocaine was 0.1 mol/dm^3^ sodium acetate-methanol (40/60, *v*/*v*), and for caffeine was 0.5 mol/dm^3^ phosphoric acid-acetonitrile-methanol (85/10/5, *v*/*v*/*v*), with a flow rate of 1 cm^3^ min^−1^. The determinations were carried out at 264 nm for ibuprofen; 254 nm for lidocaine and 272 nm for caffeine. All samples were analyzed three times. 

### 4.12. Statistical Analysis 

Results are presented as the mean ± standard deviation (SD). A one-way analysis of variance (ANOVA) was used. The significance of differences between individual groups was evaluated with Tukey’s test (α = 0.05). Statistical calculations were done using *Statistica 13* PL software (StatSoft, Kraków, Poland).

## 5. Conclusions

Due to the growing interest in natural products in the cosmetics and pharmaceutical industries, we have studied a little-known essential oil from *E. angustifolium*. The conducted research provided valuable information about the possibilities of using the essential oil obtained by us. It has been shown that EOEa is a rich source of valuable substances, mainly terpenes, which may have antioxidant and anti-microbial properties. In addition, it has also been shown that EOEa added to the medium may be an effective promoter of the skin penetration of some drugs. 

## Figures and Tables

**Table 1:** Major components of *E. angustifolium* essential oil determined with gas chromatography-mass spectrometry (GC-MS). Mean (±standard deviation), *n* = 3.

No.	Retention Time (Min)	Compound Name	Area (%)
1	9.57	Eucalyptol	3.20 ± 0.092
2	10.57	β-Linalool	4.54 ± 0.031
3	11.54	Camphor	3.90 ± 0.183
4	11.93	(+)-Isomenthol	2.42 ± 0.081
5	12.21	α-Terpineol	1.98 ± 0.072
6	12.96	(S)-Carvone	3.99 ± 0.132
7	13.49	Thymol	2.65 ± 0.177
8	13.55	Anethole	1.94 ± 0.095
9	13.65	Carvacrol	2.41 ± 0.202
10	14.39	α-Terpinyl acetate	0.53 ± 0.036
11	14.88	δ-Cadinene	0.61 ± 0.144
12	15.10	Germacradien-4-ol	1.67 ± 0.210
13	15.59	β-Caryophyllene	3.75 ± 0.234
14	16.23	γ-Cadinene	2.05 ± 0.094
15	16.75	β-Cadinene	2.11 ± 0.094
16	17.71	α-Caryophyllene oxide	8.57 ± 0.184
17	18.05	α-Caryophyllene	2.49 ± 0.397
18	18.35	β-Caryophyllene oxide	2.73 ± 0.091
19	18.50	α-Cadinol	2.60 ± 0.054
20	18.66	β- Caryophyllene	2.98 ± 0.489
21	19.94	Secalciferol	1.34 ± 0.099
	20.09		1.35 ± 0.098
22	20.27	Trimethylpentadecan-2-one	0.54 ± 0.067
23	24.72	5-Methyldocosane	14.95 ± 0.301
24	26.40	Cosanes	5.82 ± 0.304
	27.52		6.75 ± 0.335
	28.09		11.13 ± 0.779

**Table 2:** Antioxidant activity of *E. angustifolium* essential oil. Mean (±standard deviation), *n* = 3.

Antioxidant Activity	
DPPH [mg Trolox/g EOEa]	2.445 ± 0.025
RSA [%]	78.021 ± 0.755

**Table 3:** The influence of *E. angustifolium* essential oil on inhibiting of the growth of selected mold fungi (mean ± SD of the diameter of the growth inhibition zone in mm), EOEa—*E. angustifolium* essential oil.

Fungi	Concentration of EOEa (%)			
	12.5	25	50	100
*Aspergillus niger*	10.10 ± 1.00 ^a^	13.90 ± 1.80 ^b^	15.30 ± 1.50 ^c^	21.00 ± 2.20 ^d^
*Aspergillus ochraceus*	12.15 ± 1.50 ^a^	15.00 ± 1.50 ^ab^	19.10 ± 2.00 ^c^	28.30 ± 3.90 ^d^
*Aspergillus parasiticus*	9.00 ± 1.00 ^a^	12.45 ± 1.80 ^ab^	17.00 ± 2.60 ^c^	23.10 ± 2.60 ^d^
*Penicillium cyclopium*	7.80 ± 1.70 ^a^	9.00 ± 1.30 ^b^	10.00 ± 2.50 ^b^	17.10 ± 2.00 ^c^

Different letters mean significant differences between individual concentrations, *n* = 3; the analysis was performed with a one-way analysis of variance (ANOVA, Tukey’s test, α = 0.05).

**Table 4:** Skin permeation parameters for ibuprofen, lidocaine, and caffeine from emulsions with and without the *E. angustifolium* essential oil.

Compound	J_SS_, µg/cm^2^∙h	K_P_∙10^3^, cm/h	L_T_, h	D∙10^4^, cm^2^/h	K_m_	Q%_24h_
CON-IBU	26.238 ± 1.104 ^a^	2.624 ± 0.110 ^a^	1.795 ± 0.306 ^a^	2.321 ± 0.032 ^a^	0.565 ± 0.132 ^a^	2.17 ± 0.218 ^a^
EOEa-IBU	35.162 ± 1.152 ^b^	3.414 ± 0.111 ^b^	1.762 ± 0.195 ^a^	2.365 ± 0.019 ^a^	0.722 ± 0.062 ^b^	2.87 ± 0.176 ^a^
CON-CAF	16.267 ± 0.397 ^a^	1.611 ± 0.039 ^a^	1.571 ± 0.077 ^b^	2.652 ± 0.013 ^a^	0.304 ± 0.008 ^a^	1.91 ± 0.124 ^a^
EOEa-CAF	15.507 ± 3.532 ^a^	1.520 ± 0.346 ^a^	1.262 ± 0.436 ^a^	3.302 ± 0.174 ^b^	0.230 ± 0.120 ^a^	1.95 ± 0.200 ^a^
CON-LID	35.578 ± 0.732 ^a^	3.454 ± 0.071 ^a^	2.508 ± 0.037 ^a^	1.662 ± 0.024 ^a^	1.039 ± 0.006 ^a^	4.02 ± 0.685 ^a^
EOEa-LID	41.439 ± 0.239 ^b^	3.985 ± 0.022 ^a^	2.479 ± 0.064 ^a^	1.681 ± 0.046 ^a^	1.185 ± 0.025 ^a^	4.93 ± 0.353 ^b^

Jss—steady-state flux; K_P_—permeability coefficient; L_T_—lag time; D—diffusion coefficient; K_m_—skin partition coefficient; Q—the percentage of applied dose. EOEa-IBU—emulsion containing the *E. angustifolium* essential oil and ibuprofen; EOEa-LID—emulsion containing the *E. angustifolium* essential oil and lidocaine; EOEa-CAF—emulsion containing the *E. angustifolium* oil and caffeine; CON-IBU—emulsion containing only ibuprofen without the *E. angustifolium* essential oil; CON-LID—emulsion containing only lidocaine without the *E. angustifolium* essential oil; CON-CAF—emulsion containing only caffeine without the *E. angustifolium* essential oil. Different letters as ’a’ and ’b’ mean significant differences between individual emulsions (ANOVA, Tukey’s test, α = 0.05); the mean ± standard deviation SD (*n* = 3).

**Table 5:** Composition of emulsions used in pig skin penetration tests.

Ingredient	EOEa–IBU	CON–IBU	EOEa–LID	CON–LID	EOEa–CAF	CON–CAF
EOEa *	0.1	-	0.1	-	0.1	-
IBU *	0.1	0.1	-	-	-	-
LID *	-	-	0.1	0.1	-	-
CAFF *	-	-	-	-	0.1	0.1
Propylene glycol *	0.20	0.20	0.20	0.20	0.20	0.20
Biobase *	0.6	0.6	0.6	0.6	0.6	0.6
Grape seed oil *	0.20	0.20	0.20	0.20	0.20	0.20
Beeswax *	0.7	0.7	0.7	0.7	0.7	0.7
Water up to *	10	10	10	10	10	10

* The amount of components is expressed in g; EOEa—*E. angustifolium* essential oil. IBU—ibuprofen, LID—lidocaine, CAF—caffeine.

## Data Availability

The data presented in this study are available in this article.

## Supplementary Materials

The following are available online, Figure S1: The obtained the *E. angustifolium* essential oil, Figure S2: The influence of different concentrations of essential oil with *E. angustifolium* on fungi of genus *Aspergillus*, *Aspergillus niger* (A) and *Aspergillus ochraceus* (B)., Figure S3: All types of emulsions after the separation test. 1—emulsion containing the *E. angustifolium* essential oil and ibuprofen; 2—emulsion containing only ibuprofen without the *E. angustifolium* essential oil, 3—emulsion containing the *E. angustifolium* essential oil and lidocaine; 4—emulsion containing only lidocaine without the E. angustifolium essential oil; 5—emulsion containing the *E. angustifolium* oil and caffeine; 6—emulsion containing only caffeine without the *E. angustifolium* essential oil.

## Sample Availability

Samples of the compounds are available from the authors.
