# Supplementary material for: Epilobium angustifolium L. Essential Oil—Biological Activity and Enhancement of the Skin Penetration of Drugs—In Vitro Study
Source: Molecules. 2021 Nov 26;26(23):7188. doi: 10.3390/molecules26237188 (PMC8658823; doi:10.3390/molecules26237188)
Supplement: Supplementary file 1 [file molecules-26-07188-s001.zip › molecules-1457506 SM revised.pdf]

molecules

*Supplement Materials*

# *Epilobium angustifolium* L. essential oil – biological activity and enhancement of the skin penetration of drugs – *in vitro* study

**Anna Nowak [1*], Wiktoria Duchnik [2], Edyta Makuch [3], Łukasz Kucharski [1], Paula Ossowicz-Rupniewska [3], Krystyna Cybulska [4], Tadeusz Sulikowski [5] Michał Moritz [2] and Adam Klimowicz [1]**

1 Department of Cosmetic and Pharmaceutical Chemistry, Pomeranian Medical University in Szczecin, PL-70111 Szczecin, Poland; anowak@pum.edu.pl (A.N.); lukasz.kucharski@pum.edu.pl (Ł.K.); ad-klim@pum.edu.pl (A.K.)
2 Department of Pharmaceutical Chemistry, Pomeranian Medical University in Szczecin, PL-70111 Szczecin, Poland; wiktoria.duchniak@pu.edu.pl (W.D.); michal.moritz@pum.edu.pl (M.M.)
3 Department of Chemical Organic Technology and Polymeric Materials, Faculty of Chemical Technology and Engineering, West Pomeranian University of Technology, Szczecin, PL-70322 Szczecin, Poland; emakuch@zut.edu.pl (E.M.), Paula.Ossowicz@zut.edu.pl (P.O-R.)
4 Department of Microbiology and Environmental Chemistry, Faculty of Environmental Management and Agriculture, West Pomeranian University of Technology, Szczecin, PL-71434, Szczecin, Poland; Krystyna.Cybulska@zut.edu.pl (K.C.)
5 Department of General and Transplantation Surgery of Pomeranian Medical University, Pomeranian Medical University in Szczecin, PL-71252 Szczecin, Poland; tadeusz.suliowski@pum.edu.pl (T.S)
* Correspondence: anowak@pum.edu.pl; Tel.: +48-91-466-16-31

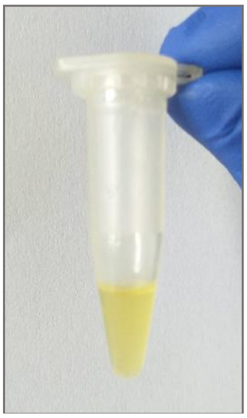

**Figure S1.** The obtained the *E. angustifolium* essential oil.

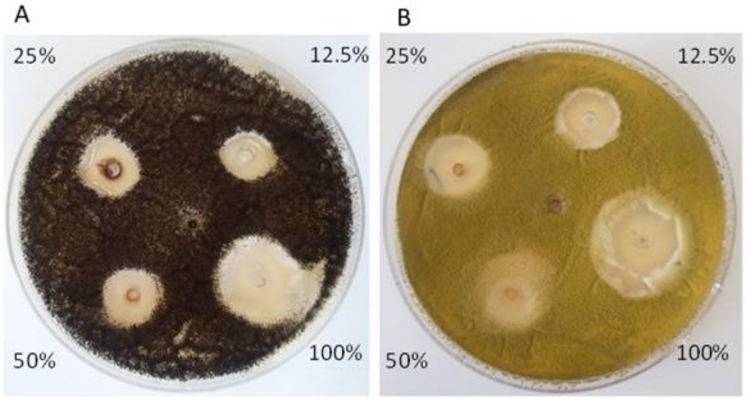


**Figure S2.** The influence of different concentrations of essential oil with *E. angustifolium* on fungi of genus *Aspergillus*, A. niger (A) and *A. ochraceus* (B).

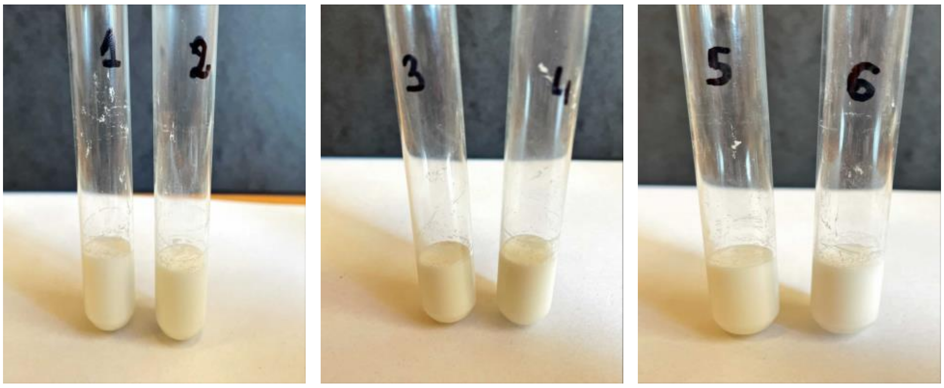


**Figure S3**. All types of emulsions after the separation test. 1 — emulsion containing the *E. angustifolium* essential oil and ibuprofen; 2 — emulsion containing only ibuprofen without the *E. angustifolium* essential oil, 3 — emulsion containing the E. angustifolium essential oil and lidocaine; 4 — emulsion containing only lidocaine without the *E. angustifolium* essential oil; 5 — emulsion containing the *E. angustifolium* oil and caffeine; 6 — emulsion containing only caffeine without the *E. angustifolium* essential oil.
